# Predicting Daily Sheltering Arrangements among Youth Experiencing Homelessness Using Diary Measurements Collected by Ecological Momentary Assessment

**DOI:** 10.3390/ijerph17186873

**Published:** 2020-09-20

**Authors:** Robert Suchting, Michael S. Businelle, Stephen W. Hwang, Nikhil S. Padhye, Yijiong Yang, Diane M. Santa Maria

**Affiliations:** 1 Faillace Department of Psychiatry and Behavioral Sciences, McGovern Medical School at The University of Texas Health Science Center at Houston (UTHealth), Houston, TX 77030, USA; 2 The University of Oklahoma Health Sciences Center, Oklahoma City, OK 73104, USA; Michael-Businelle@ouhsc.edu; 3 Oklahoma Tobacco Research Center, Stephenson Cancer Center, Oklahoma City, OK 73104, USA; 4 MAP Centre for Urban Health Solutions, St. Michael’s Hospital, Toronto, ON M5B 1W8, Canada; hwangs@smh.ca; 5 Cizik School of Nursing, University of Texas Health Science Center at Houston (UTHealth), Houston, TX 77030, USA; Nikhil.S.Padhye@uth.tmc.edu (N.S.P.); Yijiong.Yang@uth.tmc.edu (Y.Y.); Diane.M.SantaMaria@uth.tmc.edu (D.M.S.M.)

**Keywords:** youth experiencing homelessness, daily sleeping arrangement, electronic momentary assessment, machine learning, data science

## Abstract

Youths experiencing homelessness (YEH) often cycle between various sheltering locations including spending nights on the streets, in shelters and with others. Few studies have explored the patterns of daily sheltering over time. A total of 66 participants completed 724 ecological momentary assessments that assessed daily sleeping arrangements. Analyses applied a hypothesis-generating machine learning algorithm (component-wise gradient boosting) to build interpretable models that would select only the best predictors of daily sheltering from a large set of 92 variables while accounting for the correlated nature of the data. Sheltering was examined as a three-category outcome comparing nights spent literally homeless, unstably housed or at a shelter. The final model retained 15 predictors. These predictors included (among others) specific stressors (e.g., not having a place to stay, parenting and hunger), discrimination (by a friend or nonspecified other; due to race or homelessness), being arrested and synthetic cannabinoids use (a.k.a., “kush”). The final model demonstrated success in classifying the categorical outcome. These results have implications for developing just-in-time adaptive interventions for improving the lives of YEH.

## 1. Introduction

Lack of consistent sheltering options for youths experiencing homelessness (YEH) often intersects with limited access to healthcare, living wage employment, education and other unmet needs that may impede the ability to exit homelessness [1]. YEH sleep in a variety of places, ranging from the streets, places not meant for human habitation, temporarily staying with others and shelters. Youths spending the night in a shelter (SN), another person’s home (unstable housing (UH)—transient sleeping arrangements such as the home of a friend, acquaintance, partner or extended family) or less-structured locations (i.e., literally homeless (LH)—a car, street, park or abandoned building) vary in patterns of consistency in use of these different options [2].

Among a sample of YEH aged 14–24 years (*n* = 426), less than half (41%) were consistently housed over a two-year period [3]. The remaining youth used short-term (e.g., staying with a friend; 20%) or longer-term sheltering options (e.g., permanent supportive housing; 39%). In a longitudinal study of shelter-using youth (*n* = 166), 18% of first-time runaways and 34% of repeat runaways returned to the shelter within a year [4]. One recent study that included daily ecological momentary assessments (EMAs) of daily sleeping arrangements found that among YEH aged 16–22 years (*n* = 150), sleeping location changed almost daily during a one month period [5]. While the literature suggests that there is substantial variation in sheltering and transiency among YEH, little is known about the day-level predictors of those various sheltering patterns.

Previous evaluations of sheltering are often limited by analyzing cross-sectional data and have focused on measuring the lifetime, rather than day-to-day, experiences of homeless populations [6,7,8]. Given that such cross-sectional data may have been collected at a service location, this methodology may increase the likelihood that these youths will report staying in a shelter. For example, in a large, seven-city study among YEH aged 18–26 years recruited from shelters and service providing locations, YEH reported primarily sleeping at a shelter (49%) the night before participating in the survey, while 33% reported staying on the streets the night before [9]. Other studies that include youth recruited from the streets suggest that very few YEH use shelters, with rates ranging from 7% in the last three months to 21% in the last year among YEH aged 14 to 21 years [10]. Due to the variable use of shelters among this high risk and underserved population, other longitudinal methods that account for within person variance of sheltering patterns are sorely needed.

While shelters often offer a place to stay at night, onsite access to healthcare, life skills education, school success supports, workforce readiness and referrals for other services [11], there are many facilitators and barriers to accessing these shelter-based services. YEH have reported that attitudinal facilitators (e.g., the desire to extricate themselves from street life and turn their lives in a new direction) increase service utilization [12]. Yet, YEH also reported barriers to shelter access, including shelter availability and the use of restrictive definitions of homelessness that only allow prolonged street dwelling to access services [5,12]. Other reported barriers to shelter-based service utilization among YEH include negative encounters with service staff [13] and inflexible shelter rules [14]. Further, beyond structural limitations in access to resources, unstable housing presents immediate concerns related to lack of safety and potential victimization. However, understanding the factors that influence sheltering choices remains a challenge. The present research seeks to explicate the relationships between sheltering and various behavioral/environmental factors and identify the strongest predictors of sheltering option on a given night. Essentially, many factors may conceivably influence daily sheltering choices, and to date, these have seldom been explored in the literature. The present analysis uses a large-scale, data-driven approach to explore a wide set of potential factors and provide hypothesis-generating direction to this field of research. These findings would directly address the primary aims of the study: (1) to contextualize the current state of daily sheltering patterns among YEH (i.e., look beyond broad factors that implicate sheltering to find more precise, day-to-day indicators), (2) identify behavioral targets for interventions that may reduce risky sheltering (i.e., of those strongest predictors, which, if any, are specific behaviors that could be modifiable and thus targetable for intervention) and (3) inform higher-level decision making (i.e., provide direction for researchers and/or policy makers to further investigate the specific factors found here). A secondary aim of the present study is to demonstrate the application of a useful machine-learning algorithm for this research domain that can identify the strongest subset of predictors from a large set while fully accounting for multicollinearity.

### 1.1. Sexual Risk Behaviors and Sheltering

Evidence suggests that homelessness is associated with sexual risks. YEH consistently report high rates of sexual risk behaviors across studies [15,16,17]. Studies using cross-sectional designs report rates of condomless sex ranging from 40% to 70% [18] and being homeless was associated with a higher number of sexual partners [19]. A longitudinal study of EMA data indicated that condom use was much lower (25%) than indicated by self-reporting at baseline (54%) among a sample of YEH [20]. Furthermore, lacking consistent housing may lead to increased risk of trading sex for shelter [21] and exposure to sexual exploitation [22]. However, few studies have assessed the relationship between sexual activity and sheltering patterns using more granular measures that account for within person variations.

### 1.2. Role of Drug Use on Sheltering

The impact of drug use on homelessness has also been well documented across various populations, including YEH. Among YEH, greater shelter utilization has been associated with reductions in substance use [5,23,24]. YEH aged 14–24 years who used drugs were less likely to be consistently sheltered across a two-year period than those who did not use drugs [3]. In a hospital-based sample, those experiencing homelessness or unstable housing had higher rates and greater severity of alcohol and drug use than other patients seen in the emergency room [25]. Having a substance use problem is also a risk factor for failure to achieve longer-term housing stability [26]. Yet, the implications of drug use on patterns of daily sheltering over time have not yet been fully explored.

### 1.3. Gender Identity, Sexual Orientation and Sheltering

System-based and societal homophobia and transphobia act as barriers to accessing supportive services including the lack of safe, gender-affirming sheltering options [27]. Transgender and gender-nonconforming individuals often experience gender-based discrimination from service providers that may lead to disparities in access and utilization of shelters and other social services [28]. There is some evidence that cisgender women may be more satisfied with homeless youth services than cisgender men [29]. Additionally, lesbian, gay, bisexual and queer (LGBQ) youth report having more trouble finding a shelter compared to heterosexual youth [30]. Additionally, lacking an affirming, safe sheltering option may increase risk for engaging in trade sex among LGBQ youth which is one survival strategy YEH may use to secure shelter [31].

### 1.4. Using Intensive Longitudinal Assessment Methods to Identify Predictors of Sheltering Patterns

Although there have been a few studies that have investigated factors that may influence patterns of shelter use among YEH, less well understood are the factors that influence sheltering on a day-to-day basis that may be accessible via longitudinal study. EMA is currently the gold standard methodology for the measurement of real-time data in natural settings [32,33], with generally high compliance among youth across studies [34]. Several studies have shown that EMA is more accurate than self-reports that require participants to average behaviors over periods of time [35,36]. Daily diary assessments collected via EMA, which provide the compliance and accuracy benefits of the methodology, thus provide an ideal data collection method for exploring sheltering patterns and identifying predictors of daily sheltering patterns among YEH. The present exploratory secondary data analyses aimed to identify predictors of daily sheltering accommodations among YEH using demographic and daily diary items collected via EMA, including items evaluating the broad predictor classes described above. For example, the broad class of sexual risk is evaluated by items regarding any sexual activity, number of sexual partners, condom use and prostitution. The broad class of drug use is evaluated by a set of items inquiring as to any drugs used as well as alcohol and nicotine. The broad class of gender issues (identity; sexual orientation) was evaluated via questions about discrimination and sexual partners (related to above).

## 2. Materials and Methods

### 2.1. Participants and Procedures

The University’s institutional review board approved all study protocols. YEH were recruited for this study through information sessions held at drop-in centers and shelters in Houston, TX, USA between August 2015 and May 2016. Flyers were posted at the recruitment sites and contained information about the study. YEH who approached study staff during the information sessions or responded to the flyers were provided with the details of the study. YEH who expressed further interest were then assessed for eligibility. Participant accrual relied on convenience sampling and was not stratified on any variables. Invitations to participate were given to YEH who met the inclusion criteria, including having a LH or UH sheltering status, age between 18–24 years (thus meeting the state age of majority), English-speaking and a minimum 6th grade English reading comprehension level (as measured by scores ≥ 4 on the rapid estimate of adult literacy in medicine-short form) [37,38]. Reporting of sampling strategies, measures, schedule, technology used, administration, participant prompting strategy, response rate and compliance rate according to the adapted strengthening the reporting of observational studies in epidemiology checklist for reporting EMA studies [39] is done elsewhere [40]. Two respondents did not meet the criteria based on age, and no participants were deemed ineligible from the literacy test. For the purpose of study eligibility, homelessness was defined as sleeping on the streets, in a place not meant for human habitation, in a shelter, in a hotel/motel or with someone with whom they could not stay for more than 30 days (i.e., unstably housed). Participants provided written informed consent and received both a summary of the study and a copy of the informed consent document. Participants (*n* = 74) then completed an audio-assisted baseline survey on an iPad. The baseline survey took approximately 30 min to complete. Participants received a US$20 gift card for completing the baseline survey and were provided a bus ticket or METRO pass if needed. Participants could earn up to US$95 in gift cards depending on the percentage of EMAs they completed during the study period. The incentive structure was explained to all participants during the informed consent process. Youth were able to access their real-time compensation level on the study-issued smartphone. The study was in accordance with the Declaration of Helsinki and was approved by the Committee for the Protection of Human Subjects at the University of Texas Health Science Center at Houston (Project code HSC-SN-18-0501).

### 2.2. Design

The present study employed a data-driven longitudinal design to daily sheltering behavior over time in YEH. EMAs were used to collect the longitudinal data throughout the study. The EMA methodology used is similar to that developed by Shiffman, Stone and colleagues [35,36,41], and has been used by the research team in several studies [42,43,44]. The present study utilized the daily diary EMAs, which were prompted once daily 30 min after each participant’s normal waking time. The daily diary assessed events and behaviors from the previous 24 h, while random assessments provided responses relevant to a current time on a given day. Items that were included on both the random assessments and the daily diary consisted of a narrow subset concerning current affect. Merging the daily and random data would require establishing an appropriate time-lag scheme to match random assessments to the appropriate diary day. However, due to inconsistencies in response patterns across days (i.e., participants frequently skipped days), matching daily and random data were untenable in the present study. As such, data were taken exclusively from the daily diaries. However, no participants dropped out during the study, and all participants were included in the analyses on an intent-to-treat basis even if they provided fewer data points relative to others.

### 2.3. Instruments

Demographic measures and participant history were collected at baseline. Data were collected via loaned smartphones (Samsung Galaxy Light, Samsung Electronics, Seoul, South Korea; Android 4.2 operating system). Diary questions examined events and risk behaviors that occurred in the previous 24 h including sexual activity, substance use, discrimination, assault and stress. Further detail regarding each of these specific domains is provided below. A table detailing all of the predictors, including descriptive statistics and endorsement frequencies by sheltering class, is provided in Supplementary Table S1. 

#### 2.3.1. Baseline Measures

Demographic measures, including age, race/ethnicity, gender identity, sexual orientation, childhood adversities and mental illness were collected. To assess race/ethnicity, participants were asked if they identified as Black, White, Asian, Hispanic, American Indian, multiracial or something else. Participants self-reported their gender identity as cisgender man, cisgender woman, transgender man or women, gender queer, intersex, non-binary gender or something else. Sexual orientation was measured by asking youth if they identified as heterosexual, gay, lesbian, bisexual or something else. Childhood adversities were assessed using the adverse childhood experiences scale [45]. History of mental illness was assessed by asking youth if they had ever been diagnosed with attention deficit-hyperactivity disorder (ADD/ADHD), depression, bipolar disorder, psychosis, schizophrenia, oppositional defiant disorder, conduct disorder or post-traumatic stress disorder (PTSD).

#### 2.3.2. Daily Sheltering Outcome

Measurements of daily sheltering provided an initial set of nine response options: (1) relative/family home, (2) home of friend/acquaintance, (3) home of boyfriend/girlfriend/sexual partner, (4) shelter, (5) street/park/bayou/outside, (6) abandoned apartment/vaco/squat, (7) bus/metro/train, (8) car or (9) hotel/motel. Daily sheltering was coarsened to a three-level categorical variable to compare: (a) specifically staying in a homeless shelter at night (SN)**,** (b) staying in one of a set of locations categorized as literally homeless (LH) by housing and urban development point-in-time counts, and (c) sheltering considered to be unstably housed (UH). LH locations included spending the night outside, on a street, at a park, bayou, abandoned apartment, vacant apartment (colloquially referred to as a “vaco”), squat, bus, metro, train or car. UH locations included a relative/family home, the home of a friend/acquaintance, the home of a boyfriend/girlfriend/sexual partner or at a hotel/motel. The verbiage used in the survey was pilot tested with YEH in order to most accurately align with the local current lingo used among YEH to increase understandability and ease of reading.

#### 2.3.3. Domain-Specific Items

Sexual Activity—Participants were asked questions regarding their sexual behavior the previous day, including if they had sex, viewed pornography and used a condom or birth control. If they indicated “yes” to having had sex, they were asked about the number sexual partners, type of sex and gender identity of their sexual partner(s).

Substance Use—Participants were asked if they had used drugs or alcohol the previous day. If they indicated that that had used drugs, they were asked to report which drugs. Response options included marijuana, synthetic cannabinoids (e.g., locally referred to as "kush”), ecstasy (MDMA or “molly”), bath salts, sedatives, heroin, cocaine, crack, hallucinogens, cough syrup (to get high), PCP and other.

Discrimination—Participants were asked if they perceived that they were discriminated against the previous day. If they confirmed discrimination, they were asked follow-up questions regarding the perpetrator and type of discrimination. Response options for the perpetrator included a family member, boyfriend, girlfriend, stranger, acquaintance, friend, employer and other. Response options for type of discrimination included age, gender identity, race, ethnicity, religion, appearance, homelessness status and other.

Assault—Participants were asked if they had been assaulted the previous day. If they confirmed being assaulted, they were asked follow-up questions regarding the perpetrator. Response options included a family member, boyfriend, girlfriend, stranger, acquaintance, friend, employer and other.

Stress—Stress was measured via the four-item perceived stress scale PSS [46,47] (Cronbach’s alpha = 0.60), with each item assessing the perceived frequency of feeling stressed on a five-point Likert-type scale from “never” to “very often.” Participants were further asked to rate their stress on a scale from 1 (not at all stressed) to 5 (extremely stressed). If they indicated they were stressed, they were asked to endorse all the cause(s) of their stress. Response options included money, job, being pregnant, parenting, family health, not having a place to stay, personal health, safety, being hungry, boyfriend/girlfriend, friends, drugs and alcohol.

Additional Questions—Participants were asked if they worked, went to school or were arrested the previous day.

### 2.4. Procedure

After meeting eligibility criteria and providing informed consent, participants completed a battery of baseline questionnaires and were loaned a smartphone. Participants were then given instructions on how to use the smartphone to access and complete brief EMAs over the next 21-days. The application was programmed to assure that EMA prompts would only be sent during normal waking hours. A daily diary and four random sampling EMAs were completed on each day. At the end of the study, participants were compensated for their time and effort.

### 2.5. Data Analysis

#### 2.5.1. Data Processing

The categorical sheltering outcome was modeled as a function of a set of *k* = 92 predictors after recoding 30 total items: categorical predictors (*k* = 87) were dummy-coded and continuous predictors (*k* = 5) were z-scored to be on a common metric for analyses. Data consisted of 724 daily diary observations from N = 66 participants (from an overall N = 74) after listwise deletion of missingness on the sheltering outcome variable (104 observations removed). Participants provided a median 11 daily diary observations on average (ranging from 1 to 26). Missingness in the categorical predictors (~0.8% of all observations) was handled via inclusion of a “missing” categorical level, while remaining missingness at random in continuous predictors (~0.2% of all observations) was imputed using bagged imputation in the R package *caret* [48].

#### 2.5.2. Component-Wise Gradient Boosting

Component-wise gradient boosting (CGB) was used to predict the categorical daily sheltering outcome. CGB is a machine learning algorithm for building strong statistical models by iteratively combining weaker models through gradient descent [49]. The algorithm, designed as an alternative formulation of boosting algorithms [50,51], is implemented in the R statistical computing environment [52] using package *mboost* [53]. In brief, the algorithm works by additively updating the prediction of an outcome over an iterative series of models, each of which explains variability that was not explained by previous models. In each of the algorithm iterations, the single best predictor is selected to fit the updated model of the outcome. Predictors may be selected multiple times by the algorithm across iterations. The number of algorithm iterations is determined using 10-fold cross-validation, with two consequences: (1) optimized predictive performance via limited overfitting and (2), an inherent variable selection capacity, as only so many predictors may be chosen in the finite set of iterations before the algorithm terminates. Recent research has demonstrated the utility of the CGB algorithm for deriving optimized, parsimonious models of outcomes in health and behavioral sciences; examples include determinations of the best (a) inflammatory predictors of adolescent depression and anxiety [54], (b) psychosocial and genetic predictors of aggression [55] and (c) cognitive test predictors of pediatric bipolar disorder [56].

Predictors in CGB models may take multiple functional forms including linear fixed and random effects. In the present context, including a random effect for participant number allowed countenance of the correlation between observations within participant. Outcomes may be fit using a variety of statistical probability distributions and corresponding link functions; in the present analysis, the categorical outcome was fit as a multinomial variable. The relationships between predictors and a given outcome are described by parameters akin to regression coefficients. In a CGB model, these coefficients are made more robust by penalization (aka shrinkage) [57]. Complex non-penalized models with large numbers of predictors may have unstable and inflated parameter estimates due to increasing intercorrelations (collinearity) among those predictors [49]. That is, multicollinearity may result in inflated coefficients with opposite valence (i.e., positive/negative direction); penalization alleviates collinearity by decreasing the variability in estimating model coefficients. This process imposes a limit on the size of predictor coefficients, preventing such inflation and directional concerns from manifesting. A complete account of penalization/shrinkage is beyond the context of the present discussion; however, many resources are available with greater detail [58,59,60,61]. This is particularly salient to the present case, where the dummy-coded predictors are inherently related in places (e.g., each response option (yes, no, N/A) to a daily questionnaire item such as, “Who discriminated against you yesterday? (check all that apply)” will covary with the omnibus statement, “Yesterday, I felt discriminated against”). The CGB algorithm was chosen over alternative machine-learning algorithms (e.g., elastic net/lasso, random forest) for the present task due to its ability to effectively choose predictors and generate a readily interpretable model while accounting for collinearity and longitudinal/correlated data (i.e., repeated measures). The data-driven model building practiced here is not accessible through traditional generalized linear mixed models or generalized estimating equations due to the structural collinearity of the data (over and above practical issues related to model convergence due to complexity) and other machine-learning techniques (to date) lack the ability to incorporate longitudinal data in a highly interpretable framework.

#### 2.5.3. Model Interpretation

The CGB model provides coefficients with sign and magnitude to indicate the direction and strength of each retained variable’s relationship with an outcome comparison. Given the categorical outcome of the present analysis, coefficients are provided for each comparison to the reference category. These coefficients may then be exponentiated to provide odds ratios. Further, the absolute value of each coefficient is divided by the highest magnitude coefficient to provide a normalized index of variable importance, such that the strongest relationship for each comparison to the reference category is set to a value of 1.0 and each other predictor variable provides a fraction of the importance of that strongest predictor. Again, considering the multinomial outcome, the model provides ranked importance for each comparison (SN vs. LH; SN vs. UH). The normalized importance scores for each comparison are then averaged to provide an overall metric of importance across comparisons. Interpretation of these scores is essential in the application of machine-learning algorithms such as CGB, where traditional metrics for inference (i.e., standard errors and *p* values) are not accessible.

Applications of machine learning often involve a tradeoff between optimizing raw predictive performance versus parsimonious knowledge gain (i.e., maximized understanding of variable interrelationships). The present analysis focuses on the latter of these two to better understand which predictors drive patterns of daily sheltering. To that end, the CGB algorithm was run in two stages: the first stage utilized a default shrinkage parameter (nu = 0.1) as a first pass through the data [57]; this model reduced the predictor space from 92 to 35 variables. Although this provided a substantial reduction in the number of predictors retained by the model, interpretation of all 35 predictors in the context of knowledge gain was considered unwieldy. As such, the algorithm was run again through those 35 retained predictors with a shrinkage value set to 0.05 (half of the default) to further reduce the predictor space. The present manuscript focuses on the results of the second pass through the data; however, results from the first pass are included in the Supplementary Table S2 for completeness. Further, there are no established heuristics regarding the number of predictors that should be fully interpreted in these models. Rather than providing a naïve interpretation of the “top ten predictors” for a given model, we have instead chosen to primarily focus on the predictors that provided at least 25% of the averaged importance of the top-ranking variable.

The overall performance of the model was then assessed by area under the receiver operating characteristic curve (an index of the model’s ability to discriminate true positives and true negatives) and prediction accuracy (the percentage of correctly identified daily sheltering status). Accuracy is typically compared to the rate that would result from guessing the most common outcome category (the so-called no information rate). Model performance metrics were captured using the confusionMatrix function in the R package *caret* [48] and the multiclass.roc function in the R package *pROC* [62].

## 3. Results

### 3.1. Sample Characteristics

Analyses were restricted to the N = 66 participants with available data on the sheltering outcome. Participants were mostly male (62.1%), Black (65.2%), heterosexual (78.8%) and unemployed (83.3%). Male participants had slightly higher mean age (male: 21.2 (SD = 1.9); female = 20.8 (SD = 2.0)). Male and female participants were represented across racial categories (male: 68.3% Black; female: 62.5% Black, each relative to other race), orientation (male: 87.87% heterosexual; female: 62.5% heterosexual) and employment status (male: 17.1% employed; female: 16.7% employed). The present non-randomized sample was largely representative of the YEH population in the Houston metropolitan area.

### 3.2. Sheltering Characteristics

Across the 724 observations, participants more often reported their previous night’s sleeping arrangements as UH (*n* = 362, 50.0%) versus LH (*n* = 262, 36.2%) or SN (*n* = 100, 13.8%). The locations characterized as UH were staying at a relative’s house or in the family home (*n* = 107/724; 14.8%), staying with a friend or acquaintance (*n* = 102/724; 14.1%), staying in the home of a boyfriend, girlfriend or sexual partner (*n* = 97/724; 1.31%) or staying in a hotel/motel (*n* = 56/724; 7.7%). The most frequent LH locations were staying on the street, in a park or near a bayou (*n* = 135; 18.6%), staying in an abandoned apartment, vaco or squat (*n* = 94; 13.0%), staying in a bus, metro or train (*n* = 24; 3.3%) or staying in a car (*n* = 9; 1.2%).

Participants experienced various possible combinations of shelter types during the study. Eighteen participants reported each type at least once. Of these, 125 observations were UH, 70 observations were LH, and 37 observations were SN. Twenty-four participants reported a combination of two types: 20 reported UH and LH, three reported UH and SN, and one reported LH and SN. The first of these hybrids were slightly less characterized by UH (119 observations) than LH (123 observations), the second hybrid was nearly evenly split between UH and SN (22 and 21 observations, respectively), and the third hybrid consisted of one LH and one SN observation each. Finally, 13, 7 and 4 participants reported only one type (respectively, 96 UH, 68 LH and 41 SN observations).

### 3.3. Component-Wise Gradient Boosting

The CGB algorithm was used to derive an optimized model fitting the categorical sheltering variable whereby SN was compared to both LH and UH using a set of 92 predictors. With the default shrinkage parameter nu = 0.1, tuning the optimal number of boosting iterations by 10-fold cross-validation resulted in a model featuring 35 predictors. Penalized coefficients, odds ratios, normalized importance scores, and raw endorsement frequency by category for the selected predictors are included in Supplementary Table S2. This model of 35 predictors was further reduced by another pass through the algorithm with the shrinkage parameter set to half of the default (nu = 0.05). Tuning the second pass through the remaining predictors resulted in a model featuring 15 predictors (Table 1).

Interpreting a given predictor in Table 1 follows from understanding the odds ratios, normalized importance and raw endorsement frequencies. For example, the predictor with the highest average normalized importance was endorsing the response option, “Not having a place to stay” to the item, “What were you stressed about?” (OR_LH_ = 1.37; OR_UH_ = 0.90). These odds ratios may be interpreted such that endorsing the “Not having a place to stay” option was associated with a 37% increase in the odds of experiencing a LH evening compared to a SN evening and a corresponding 10% decrease in the odds of experiencing a UH evening compared to a SN evening. Odds ratios were calculated in the present study by exponentiating the raw penalized coefficients reported by the tuned algorithm. Subsequently examining the frequency of endorsement for each outcome category aids interpretation: the “Not having a place to stay” response option was endorsed more than twice as often for LH than UH nights and approximately five times more often than SN.

The relative strength of the various predictor relationships with the outcome may be investigated via further consideration of the normalized importance scores. The predictor with the second-highest normalized average importance was endorsing the response option, “Yes” to the item, “Were you arrested yesterday?” (OR_LH_ = 0.87; OR_UH_ = 1.33). These odds ratios correspond to a 13% decrease in the odds of a LH and a 33% increase in the odds of a UH, each relative to a SN. The importance scores (ordered in Table 1 by normalized average importance, high to low) provide additional detail: for the LH versus SN comparison, the importance was 43.8% that of the strongest predictor and for the UH versus SN comparison, the predictor yielded the top rank in importance; subsequent averaging of these importance scores demonstrated an average normalized importance of 99.5%. In essence, this predictor provided almost the same amount of overall predictive value to the model as the top ranking predictor (not having a place to stay), with the understanding that the variable contributes more to understanding the UH versus SN comparison relative to the LH versus SN comparison. The frequency of endorsements support this interpretation, with substantially higher frequencies reported for the UH nights relative to the other categories.

Eight additional predictors provided at least 25% of the average normalized importance of the top predictor; these are described here with predicted probabilities relative to a SN except where otherwise specified. These predictors included indicating that a friend had discriminated against the participant yesterday (OR_LH_ = 1.30; OR_UH_ = 0.89), responding that race was the primary reason for experiencing discrimination (OR_LH_ = 1.28; OR_UH_ = 0.92), using synthetic cannabinoids (a.k.a., “kush”; OR_LH_ = 1.25; OR_UH_ = 0.93), reporting having had sex with an unspecified other person (i.e., not a significant other or a prostitute; OR_LH_ = 0.99; OR_UH_ = 1.14), receiving verbal abuse (OR_LH_ = 1.08; OR_UH_ = 0.96), not responding to the item regarding having worked yesterday (OR_LH_ = 1.01; OR_UH_ = 1.12), being physically assaulted (i.e., hit/ punched/slapped/kicked; OR_LH_ = 0.99; OR_UH_ = 0.11) and stress about parenting (OR_LH_ = 0.99; OR_UH_ = 0.12). Additional predictors in the model may be interpreted in similar fashion, but do not provide as much predictive utility. The relative importance ascribed to the remaining five predictors selected by the reduced model may be given attention accordingly.

Weaker predictors of the sheltering outcome that did not meet the 25% importance threshold of the present study deserve accordingly lower, but still some, attention here, given that the tuned algorithm chose to retain them (especially than the 77 predictors the algorithm did not retain). The remaining predictors of a LH night were reporting stress about hunger, receiving discrimination from an unspecified other, not asking a sex partner if they wanted to have sex before it happened each time and receiving discrimination due to being homeless. The remaining predictor of a UH night was an affirmative response to having worked yesterday. Percentage changes in the odds of a LH or UH night did not exceed 5% for any of these relatively less important predictors.

Model performance metrics indicated that the algorithm provided daily shelter status classification accuracy of 79.9%, a significant *(p <* 0.001) improvement over the no information rate of 50.0% (represented by choosing LH, the most frequent category, for each prediction). The algorithm more readily distinguished the UH locations (92.0%) than the LH locations (71.8%) or the shelter (58.0%). The algorithm’s overall ability to distinguish LH from UH was given by the multiclass AUC = 0.92.

## 4. Discussion

The present study applied data science techniques to three weeks of intensive longitudinal data to predict different sheltering patterns among YEH using innovative methodology. Sheltering patterns varied within and across participants over the study period indicating substantial transiency among YEH. Although shelters are primed for assisting youth in accessing needed resources and services, only one in five nights within the study period were collectively spent in a shelter. YEH utilize shelters less commonly than other types of services such as drop-in centers [12,63] or staying temporarily with others. Consistent with the literature [2,3], this signals the need to potentially broaden the definition of homelessness and/or modify point-in-time counting methodologies to account for variations in sheltering patterns among YEH and reduce the risk of undercounting disconnected youth in need of services who may be UH during a point-in-time count, rather than LH [64]. Findings from this study identified predictors of shelter use and literal homeless nights that should be considered by service providers. These findings can be used to inform policies that support low-barrier access to shelters and homeless services.

The results of the present study are summarized in Table 1. Interpretative statements here directly follow from the coefficients, importance measurements, and endorsement frequencies described for each retained predictor. Generally, the probability of a SN fell between the probability of either a LH or a UH night, i.e., most predictors followed a pattern of lower-to-higher probabilities of LH > SN > UH or LH < SN < UH. For example, endorsing the “Not having a place to stay” response option of the question, “What were you stressed about yesterday?” followed the former pattern of being more likely to experience literal homelessness than use a shelter or find unstable housing. Responding “yes” to the question, “Were you arrested yesterday?” followed the latter of being more likely to experience unstable housing, i.e., a night in jail. One exception was noted to this pattern, such that not responding to the item, “I worked yesterday” was related to higher probabilities for both LH and UH relative to SN.

In the present study, stress related to not having a place to stay, being arrested, experiencing discrimination (particularly due to race) and using synthetic cannabinoids were the strongest predictors of not staying in a shelter on a given night (> 50% normalized importance). This may be driven by substance use policies in the shelters, spending the night in jail and being denied access to a shelter related to perceived discrimination. Additional predictors demonstrating a substantial contribution to the model (those between 25% and 50% normalized importance) included having sex with an unspecified other (i.e., not a significant other or a prostitute) and being physically (i.e., hit, slapped, punched or kicked) or verbally abused. This may indicate that youth who secure unstable housing may be doing so in exchange for sex and violence on those nights. Youth who are parenting may perceive unstable housing to be safer than shelters, thus increasing their use of unstable housing. Shelters often highly encourage and/or require youth to be actively working or seeking employment, which may lead to less shelter use for those who are not working.

Several of the strongest predictors may be directly related to the broad classes of factors related to homelessness that were discussed in Section 1.1–1.3 in this manuscript. Regarding sexual activity, having sex with an unspecified individual was related to a 14% increase in the odds of a UH night. Drug use was captured by synthetic cannabinoid (“kush”) use (+ 25% increased odds of a LH night). Factors directly related to gender identity and sexual orientation were not identified by the algorithm; however, the first pass through the algorithm (Supplementary Table S2) identified sex with a non-binary partner as related to increased odds (+10%) of a LH night (this variable was likely not selected by the final model due to low frequencies of endorsement). Further studies are needed to disentangle these phenomena. Research methods that merge geographical data, longitudinal data, and qualitative interviews may enhance our understanding the drivers of sheltering patterns. Such methodology has been instructive in furthering our understanding of geographical connections to substance use [65].

Synthetic cannabinoid (“kush”) use was found to strongly predict the nights that youth spent on the streets relative to SN or UH nights. However, drug use was less predictive of staying in a shelter compared to any other place. The literature clearly supports that drug use is associated with less shelter use [5,23,24] and less housing stability overall [3]. The findings from this study suggest that on the days that YEH use synthetic cannabinoids, they are more likely to be LH. More research is needed to determine best strategies for sheltering youth who use substances both within emergency shelters and in more permanent housing options. While substance use was less likely on the nights one used a shelter, it is unclear whether substance use follows the inability to secure temporary shelter or if youth are denied the ability to stay in a shelter due to using substances. Event-based assessments inquiring about sheltering attempts would increase our understanding of the critical points that lead to LH nights among substance using YEH. Shelter-based substance using spaces have been explored as a way to increase safety and reduce overdose among homeless populations in Canada [66] and may improve rates of shelter use.

Many predictors were not selected by the algorithm in the present study; the final model only retained 15 of 92 predictors (thus discarding the 77 others). The non-retained predictors included those related to nicotine and alcohol use, other stressors (e.g., money, job, personal safety), aspects of sexual activity (type, partner’s gender identity, securing active sexual consent, condom use), school attendance, several other types of discrimination (e.g., age, gender identity) and sources (e.g., family member, friend) and other assault types (e.g., robbed, held against will) and sources (e.g., family member, significant other). This may indicate that, while still all too common experiences for YEH, these factors may not be as strongly related to where one stays on a given night as much as the other predictors. However, it is important to explore these phenomena further in larger studies using mixed methods to improve our understanding of sheltering patterns and inform interventions that address barriers to sheltering and prevention efforts needed to keep unstably housed youth safe. Further, the present

### 4.1. Implications and Significance of the Present Findings

This is the first study to use longitudinal data and techniques from data science to explore patterns of sheltering and to predict the likelihood of utilizing a shelter or unstable housing among a high-risk, hard-to-reach, population of youth experiencing homelessness. The longitudinal and applied machine-learning methodologies used here are potentially applicable to other hard-to-reach populations and have been used to predict other risk behaviors such as sexual activity and substance use that vary across days, occur with frequency and are potentially affected by real-time factors [20,40].

Although the present study does not evaluate causality and generalizability may be limited to YEH that interface with shelter and drop-in service locations, the present study was able to isolate a small, parsimonious set of factors demonstrating the strongest relationship to daily sheltering. Moreover, the methodology used here provided an index of the relative importance of each predictor in the model, in essence ranking the predictors. Although we may have generally expected the direction of influence for each predictor, understanding the relative contribution of the predictors provides considerable value (e.g., racial discrimination, particularly by a friend, is more predictive of a LH night than stress about hunger). Further, given that the algorithm focused attention on 15 predictors while discarding 77 provides an optimized set of variables for further investigation. In essence, future efforts may place more value on targeting interventions at these predictors than the non-retained predictors, particularly those with the strongest relationship to the outcome.

This study deepens our understanding of the variation and transiency in sheltering patterns as well as suggesting that it is possible to predict days when youth are less likely to access the relative safety of emergency shelters. This study adds valuable information to the literature regarding the aforementioned broad factors related to homelessness as well as a starting point for investigating specific predictors related to daily sheltering in future studies. Moreover, with this data, it may be possible to develop just-in-time messaging and alerts that can disrupt the progression from drug use to unstable or literal homeless nights and encourage safer sexual practices on nights when youth are unstably housed. Further research is needed to inform violence prevention efforts for youth experiencing unstable housing. Finally, findings from this study indicate there may be a need for location specific resource navigation to assist youth in finding safer sheltering options and seeking alternatives to unstable housing that may increase the risk of experiencing violence.

### 4.2. Limitations and Future Directions

One limitation of the present research lies in the confusion that may arise from the disparate definitions of UH, LH and SN have arisen in the study of housing instability over time. The present research relies on a distinction between UH and LH that has been described as precariously or marginally housed by some [67] but largely concurs with research suggesting that housing instability lacks a fundamental, standard definition [68,69] irrespective of authoritative criterions (e.g., the HEARTH Act in the United States). The present research also has methodological constraints: although machine learning allows for exploration of all measured potential predictors for an outcome, (e.g., sheltering), the findings may not reflect other possible factors that may influence sheltering but were not measured. While the daily survey was based on extensive formative research [70,71,72], this particular outcome of sheltering patterns was not a primary research question. Nevertheless, using longitudinal data and machine learning methodologies to assess sheltering patterns and predictors is a novel approach that accounts for large variabilities within and across participants. Of note, these data collection approaches are a class of relatively new methods. As a result, several of the measures used to assess these factors have not yet been psychometrically validated. Further, the response patterns of the participants necessitated a coarsening of the available data to focus strictly on the daily diary observations. This limitation inherently restricts the granularity of the predictions possible by the algorithm; however, it may be somewhat tempered by the wide predictor set that was available on the daily diary observations. Data temporality is another limitation, as youth provided retrospective reports of sheltering behavior on the previous day. Therefore, we cannot conclude whether these predictors (e.g., drug use, sexual behaviors) lead to sheltering choices or were a byproduct/consequence of that sheltering choice. The current study does not evaluate causality.

Another limitation is the sampling strategy used in this study. While the use of frequent assessments of sheltering patterns over a period of time is an improvement from cross sectional designs, the participants were recruited from service locations and were compensated for their participation. Therefore, the results may not be generalizable to youth who do not interface with shelter or drop-in center services. Further studies should include youth recruited from the streets. In addition, to the extent that disparate samples are different from the present sample, not all of the results may generalize to YEH. For example, synthetic cannabinoids were particularly salient to the present sample at the time of data collection; other samples may be more influenced by other drugs (or none). Other predictors may similarly be influenced by sampling concerns. Finally, it is important to conduct subsequent studies to determine the reproducibility of the patterns that emerged in this study. Future research should investigate the extent to which sample characteristics moderate the relationships between these predictors and sheltering.

## 5. Conclusions

EMA allows for the high-compliancy and accurate capture of daily diary longitudinal data that is primed for assessing variable outcomes and factors related to sheltering. Policy makers, health and social service providers, and researchers should consider the implications of these findings to improve shelter access and availability to youth experiencing homelessness. Further research is needed to inform the development of and testing of apps that can send relevant and timely messages regarding available sheltering options and/or address the high risk variables that may drive nights youth stay on the streets or in unstable and unsafe housing situations.

## Figures and Tables

**Table 1:** Component-wise gradient boosting—reduced model. Outcome predicted categories: literally homeless (LH) or unstable housing (UH) as opposed to sheltered night); OR > 1.0 indicate greater odds of a LH or UH night relative to a SN, while OR < 1.0 indicate lower odds relative to a SN. No shading—odds UH > SN > LH; light gray—odds LH > SN > UH; dark gray—UH > LH > SN. Results ordered by normalized average importance (high to low).

Predictor—Response Option	Odds Ratio (LH)	Odds Ratio (UH)	Normalized Importance (LH)	Normalized Importance (UH)	Normalized Average Importance	Frequency Endorsed LH Nights	Frequency Endorsed UH Nights	Frequency Endorsed SN Nights
What were you stressed about?—Not having a place to stay	1.38	0.90	100.0%	37.3%	100.0%	104	47	21
Were you arrested?—Yes	0.87	1.33	43.8%	100.0%	99.5%	4	14	1
Who discriminated against you yesterday?—Friend	1.30	0.89	81.9%	41.7%	89.3%	12	3	0
What was the main reason(s) for the discrimination that you experienced yesterday—Race	1.28	0.92	76.4%	29.5%	77.1%	21	6	0
Yesterday, I used the following substances—“Kush”	1.25	0.93	70.0%	27.7%	71.0%	20	5	0
Who did you have sex with yesterday—Other	0.99	1.14	4.5%	46.2%	34.1%	20	27	1
Yesterday, I was [Assaulted]—Verbally abused	1.08	0.96	25.2%	15.5%	29.3%	30	12	5
I worked yesterday—No response	1.01	1.12	1.8%	40.6%	28.4%	50	91	7
Yesterday, I was [Assaulted]—Physical (hit/slapped/punched/kicked)	0.99	1.11	3.5%	38.1%	28.0%	30	67	9
What were you stressed about?—Parenting	0.99	1.12	2.2%	39.4%	27.9%	31	52	4
What were you stressed about?—Hunger	1.05	0.96	15.2%	13.7%	20.6%	73	37	20
I worked yesterday—Yes	0.99	1.03	3.2%	9.3%	8.6%	198	240	86
Who discriminated against you yesterday?—Other	1.02	0.99	5.9%	2.4%	6.0%	24	7	1
Did you ask the person if they wanted to have sex before it happened each time?—No	1.01	0.99	4.3%	2.3%	4.7%	26	13	3
What was the main reason(s) for the discrimination that you experienced yesterday—Homeless	1.01	0.99	3.9%	2.5%	4.6%	21	4	2

## Supplementary Materials

The following are available online at https://www.mdpi.com/1660-4601/17/18/6873/s1. The supplemental material for the present manuscript includes two tables. Table S1 includes a full account of the candidate predictors entered into the component-wise gradient boosting algorithm. Table S2 describes the results from the first pass through the algorithm. These retained 35 predictors were then entered into a second pass through the algorithm with a more intense penalty to maximize parsimony.
