# Supplementary material for: Predicting Daily Sheltering Arrangements among Youth Experiencing Homelessness Using Diary Measurements Collected by Ecological Momentary Assessment
Source: Int J Environ Res Public Health. 2020 Sep 20;17(18):6873. doi: 10.3390/ijerph17186873 (PMC7558709; doi:10.3390/ijerph17186873)
Supplement: Supplementary file 1 [file ijerph-17-06873-s001.pdf]

Supplementary Material

The supplementary material for the present manuscript includes two tables. Table S1 includes a full account of the candidate predictors entered into the component-wise gradient boosting algorithm. Table S2 describes the results from the first pass through the algorithm. These retained 35 predictors were then entered into a second pass through the algorithm with a more intense penalty to maximize parsimony.

Table S1. Full Predictor Set - Daily Item Endorsement and Descriptive Statistics.

Daily Question	Variable Level	Not Endorsed	Endorsed	Endorsement Percentage	Endorsed by UH	Endorsed by LH	Endorsed by SH
Discriminated Against	Yes (vs. No)	657	67	9.25%	33	31	3
Discrimination - Reason	Age	698	26	3.59%	15	11	0
Discrimination - Reason	Gender	707	17	2.35%	7	10	0
Discrimination - Reason	Race	697	27	3.73%	6	21	0
Discrimination - Reason	Ethnicity	711	13	1.80%	4	9	0
Discrimination - Reason	Religion	710	14	1.93%	3	10	1
Discrimination - Reason	Height or Weight	711	13	1.80%	4	9	0
Discrimination - Reason	Other Aspect of Appearance	712	12	1.66%	3	9	0
Discrimination - Reason	Physical Disability	718	6	0.83%	3	3	0
Discrimination - Reason	Sexual Orientation	715	9	1.24%	0	8	1
Discrimination - Reason	Being A Smoker	715	9	1.24%	4	5	0
Discrimination - Reason	Being Homeless	697	27	3.73%	4	21	2
Discrimination - Reason	Being Poor	700	24	3.31%	4	21	2
Discrimination - Reason	Nonspecified Other	716	8	1.10%	4	3	1
Discrimination - Source	Boyfriend / Girlfriend	715	9	1.24%	6	3	0
Discrimination - Source	Family Member	707	17	2.35%	13	4	0
Discrimination - Source	Friend	709	15	2.07%	3	12	0
Discrimination - Source	Nonspecified Other	692	32	4.42%	7	24	1
Assault - Type	Physical (Hit, Slapped, Punched, Kicked)	618	106	14.64%	67	30	9
Assault - Type	Robbed	697	27	3.73%	17	7	3
Assault - Type	Verbally Abused	677	47	6.49%	12	30	5
Assault - Type	Held Against Will	718	6	0.83%	2	3	1
Assault - Source	Boyfriend / Girlfriend	687	37	5.11%	21	13	3
Assault - Source	Friend / Stranger / Aquaintance	687	37	5.11%	18	14	5
Assault - Source	Family Member	705	19	2.62%	9	10	0

Assault - Source	Nonspecified Other	675	49	6.77%	28	20	1
Police - Interacted With	Yes (vs. No)	666	58	8.01%	35	20	3
Police - Arrested By	Yes (vs. No)	705	19	2.62%	14	4	1
Sex - Had Sex Yesterday	Yes (vs. No)	589	135	18.65%	74	53	8
Sex - Partner	Boyfriend / Girlfriend	631	93	12.85%	53	34	6
Sex - Partner	Prostitute	718	6	0.83%	4	1	1
Sex - Partner	Other	676	48	6.63%	27	20	1
Sex - Type	Oral	659	65	8.98%	35	26	4
Sex - Type	Vaginal	617	107	14.78%	61	40	6
Sex - Type	Anal	704	20	2.76%	11	8	1
Sex - Partner Gender	Male	668	56	7.73%	28	25	3
Sex - Partner Gender	Female	647	77	10.64%	45	28	4
Sex - Partner Gender	Non-Binary	717	7	0.97%	4	2	1
Sex - Planned	Yes (vs. No)	648	76	10.50%	47	26	3
Sex - Asked Partner To Have	Yes (vs. Other Options)	644	80	11.05%	47	26	3
Sex - Asked Partner To Have	No (vs. Other Options)	682	42	5.80%	13	26	3
Sex - Asked Partner To Have	I Don't Know (vs. Other Options)	711	13	1.80%	8	4	1
Sex - Permission Given	Yes (vs. Other Options)	633	91	12.57%	49	37	5
Sex - Permission Given	No (vs. Other Options)	687	37	5.11%	21	14	2
Sex - Permission Given	I Don't Know (vs. Other Options)	717	7	0.97%	4	2	1
Sex - Condom Used	Never (vs. Other Options)	663	61	8.43%	34	24	3
Sex - Condom Used	Sometimes (vs. Other Options)	684	40	5.52%	25	14	1
Sex - Condom Used	Always (vs. Other Options)	690	34	4.70%	15	15	4
Sex - Traded Sex	No (vs. Other Options)	706	18	2.49%	64	46	7
Sex - Traded Sex	Yes (vs. Other Options)	607	117	16.16%	10	7	1
Sex - Pornography Used	Yes	576	148	20.44%	73	64	11

	(vs. No)						
Stress - Stressor	Money	444	280	38.67%	118	122	40
Stress - Stressor	Job	510	214	29.56%	89	86	39
Stress - Stressor	Pregnancy	666	58	8.01%	44	10	4
Stress - Stressor	Parenting	637	87	12.02%	52	31	4
Stress - Stressor	Family Member Health	658	66	9.12%	32	30	4
Stress - Stressor	Not Having A Place To Stay	552	172	23.76%	47	104	21
Stress - Stressor	Personal Health	632	92	12.71%	38	44	10
Stress - Stressor	Personal Safety	631	93	12.85%	32	50	11
Stress - Stressor	Hunger	594	130	17.96%	37	73	20
Stress - Stressor	Boyfriend / Girlfriend Issues	584	140	19.34%	79	53	8
Stress - Stressor	Friends	632	92	12.71%	36	41	15
Stress - Stressor	Drugs	656	68	9.39%	24	38	6
Stress - Stressor	Alcohol	696	28	3.87%	13	15	0
Stress - Stressor	Other Stressor	609	115	15.88%	50	38	27
Nicotine - Products Used	Cigarettes	424	300	41.44%	163	119	18
Nicotine - Products Used	Chewing Tobacco / Snuff	703	21	2.90%	13	7	1
Nicotine - Products Used	E-Cigarettes	692	32	4.42%	22	10	0
Nicotine - Products Used	Hookah	694	30	4.14%	18	9	3
Nicotine - Products Used	Nicotine Replacement (Gum / Lozenge / Patch)	686	38	5.25%	28	7	3
Nicotine - Products Used	Cigars / Cigarillos	634	90	12.43%	47	40	3
Nicotine - Smoking Status	Current Smoker (vs. Non-smoker)	117	607	83.84%	301	211	95
Nicotine - Smoking Amount	Less than 10 Cigarettes/Day (vs. Other Options)	333	391	54.01%	189	120	82
Nicotine - Smoking Amount	More than 10 Cigarettes/Day (vs. Other Options)	508	216	29.83%	112	91	13
Substances Used	Kush	699	25	3.45%	5	20	0
Substances Used	Marijuana	694	30	4.14%	11	18	1

Substances Used	Ecstasy	586	138	19.06%	85	46	7
Substances Used	Sedatives / Pain Pills	707	17	2.35%	10	7	0
Alcohol - Drank Yesterday	Yes	677	47	6.49%	20	24	3
Alcohol - Drank Yesterday	No	194	530	73.20%	252	188	90
Alcohol - Drank Yesterday	Did Not Respond	577	147	20.30%	90	50	7
Work - Engaged In	Yes	200	524	72.38%	240	198	86
Work - Engaged In	No	672	52	7.18%	31	14	7
Work - Engaged In	Did Not Respond	576	148	20.44%	91	50	7
School - Attendance	Yes	197	527	72.79%	234	202	91
School - Attendance	No	675	49	6.77%	37	10	2
School - Attendance	Did Not Respond	576	148	20.44%	91	50	7
		**Mean**	**SD**				
Perceived Stress Scale Total	Sum of Four 5-Point Likert-Type Items	11.4	4.14				
Sex - Number Times	1 to 4+	0.4	0.96				
Sex - Number Partners	1 to 10+	0.54	1.48				
Stress - Amount	1 (Not at all) to 5 (Extremely)	2.71	1.46				
Alcohol - Number Drinks	0 to 8+	0.36	1.44				

Table S2. Component-wise Gradient Boosting Model – First Pass (35 Retained Predictors).

Outcome predicted category = Literally Homeless or Unstable Housing (as opposed to Sheltered Night); OR > 1.0 indicate greater odds of a LH or UH night relative to a SN, while OR < 1.0 indicate lower odds relative to a SN. No shade: odds UH > SN > LH. Light gray: odds LH > SN > UH. Dark gray: UH > LH > SN. Table is ordered such by normalized average importance (high to low).

Full Variable	Odds Ratio (LH)	Odds Ratio (UH)	Normalized Importance (LH)	Normalized Importance (UH)	Normalized Average Importance	Frequency Endorsed on LH Nights	Frequency Endorsed on UH Nights	Frequency Endorsed on SH Nights
Who did you have sex with yesterday - Prostitute	0.379	1.677	99.3%	49.4%	100.0%	1	4	1
Who discriminated against you yesterday? - Friend	2.660	0.655	100.0%	40.4%	94.2%	12	3	0
Did you ask the person if they wanted to have sex before it happened each time? - No	2.262	0.581	83.4%	51.9%	91.4%	26	13	3
Who did you have sex with yesterday - Other	0.860	2.847	15.5%	100.0%	80.5%	20	27	1
Yesterday, I used the following substances - Kush	2.165	0.775	79.0%	24.4%	69.1%	20	5	0
Who discriminated against you yesterday? - Boyfriend/Girlfriend	0.814	2.020	21.1%	67.2%	61.1%	3	6	0
Yesterday, I used the following substances - Sedatives/Pain Pills	0.797	1.646	23.1%	47.6%	48.7%	7	10	0
What were you stressed about? - Parenting	0.947	1.778	5.6%	55.0%	42.4%	31	52	4
I worked yesterday – Yes	0.839	1.526	18.0%	40.4%	40.2%	198	240	86
What were you stressed about? - Hunger	1.255	0.706	23.2%	33.3%	38.6%	73	37	20
What were you stressed about? - Not Having A Place To Stay	1.487	0.860	40.5%	14.4%	36.8%	104	47	21
What was the main reason(s) for the discrimination that you experienced yesterday - Disability	0.849	1.405	16.8%	32.5%	33.9%	3	3	0
Were you arrested?	0.854	1.372	16.1%	30.2%	31.9%	4	14	1

- Yes								
What was the main reason(s) for the discrimination that you experienced yesterday - Race	1.419	0.906	35.8%	9.4%	30.2%	21	6	0
Yesterday, I was…[Assaulted] - Verbally Abused	1.228	0.881	21.0%	12.1%	22.3%	30	12	5
Who assaulted you yesterday? - Family Member	1.306	0.947	27.3%	5.2%	21.6%	10	9	0
What were you stressed about - Other	0.844	1.136	17.3%	12.2%	19.9%	38	50	27
What gender was your partner(s) yesterday? - Non-binary	1.102	0.835	10.0%	17.2%	18.6%	2	4	1
What type of sex did you have? - Oral	1.188	0.924	17.6%	7.6%	16.9%	26	35	4
What were you stressed about - Alcohol	0.962	1.218	4.0%	18.9%	15.9%	15	13	0
Did you give your permission to have sex before it happened each time? - No	0.915	1.136	9.0%	12.1%	14.5%	14	21	2
Yesterday, I was…[Assaulted] – Physical (Hit/Slapped/Punched/Kicked)	0.999	1.184	0.1%	16.2%	11.4%	30	67	9
What type of sex did you have? – Anal	0.944	1.106	5.9%	9.6%	10.6%	8	11	1
How stressed were you yesterday? (1 = "Not At All Stressed" to 5 = "Extremely Stressed")	1.054	0.908	5.4%	9.2%	10.0%	--	--	--
How many cigarettes did you smoke yesterday - More Than 10	1.086	1.023	8.5%	2.2%	7.1%	91	112	13
Who discriminated against you yesterday? - Family Member	0.974	1.076	2.7%	7.0%	6.7%	4	13	0
Who assaulted you yesterday? - Friend	1.033	0.943	3.3%	5.6%	6.1%	14	18	5
How many cigarettes did you smoke yesterday - Fewer Than 10	0.969	1.051	3.2%	4.8%	5.5%	120	189	82
Did you give your permission to have sex before it happened each time? - Yes	1.058	0.993	5.7%	0.7%	4.3%	37	49	5
Personal Stress Scale - Total Score	1.010	0.958	1.0%	4.1%	3.5%	--	--	--
Select all nicotine products you used yesterday - E-Cigarettes	0.993	1.032	0.7%	3.0%	2.6%	10	22	0
What was the main reason(s) for the discrimination that you experienced yesterday - Homeless	1.025	0.986	2.5%	1.3%	2.6%	21	4	2
Select all nicotine products you used yesterday - Cigar	1.034	0.996	3.4%	0.4%	2.5%	40	47	3

Who discriminated against you yesterday? - Other	1.018	0.994	1.8%	0.6%	1.6%	24	7	1
I worked yesterday	1.006	1.196	0.0%	0.0%	0.0%	50	91	7
